# Verification of Commercial Near-Infrared Spectroscopy Measurement and Fresh Weight Diversity Modeling in Brix% for Small Tomato Fruits with Various Cultivars and Growth Conditions

**DOI:** 10.3390/s23125460

**Published:** 2023-06-09

**Authors:** Masazumi Ino, Eiichi Ono, Yo Shimizu, Kenji Omasa

**Affiliations:** Faculty of Agriculture, Takasaki University of Health and Welfare, 54 Nakaorui-machi, Takasaki 370-0033, Gunma, Japanshimizu-y@takasaki-u.ac.jp (Y.S.)

**Keywords:** cherry tomato, currant tomato, non-destructive measurement

## Abstract

The use of non-destructive commercial near-infrared (NIR) spectroscopy to estimate Brix% was verified using all samples of cherry tomato ‘TY Chika’, currant tomato ‘Microbeads’, and the M&S or market-purchased and supplemental local source tomatoes. Additionally, the relationship between fresh weight and Brix% of all samples was examined. These tomatoes had a diversity of cultivars, growing methods, harvest timing, and production locations and varied widely from 4.0% to 14.2% for Brix% and 1.25 g to 95.84 g for fresh weight. Regardless of the diversity of all samples, it was revealed that the refractometer-based Brix% (y) was practically estimated from the NIR-derived Brix% value (x) using a relationship of y = x (RMSE = 0.747 Brix%) after only a one-time calibration for the NIR spectrometer offset. An inverse relationship between fresh weight and Brix% could be modeled using a hyperbolic curve fit, and the model showed an R^2^ of 0.809 except for ‘Microbeads’. The Brix% of ‘TY Chika’ was highest on average (9.5%) and had a large difference from 6.2 to 14.2% among the samples. Data distribution of cherry tomato groups such as ‘TY Chika’ and M&S cherry tomatoes was closer, indicating a roughly linear correlation between fresh weight and Brix%.

## 1. Introduction

The sugar content of tomato fruits varies depending on cultivars, growing methods, and harvest timing [1,2,3]. Fructose and glucose are the dominant sugar components in tomato and cherry tomato fruits, and they contain less sucrose [4]. Taste preferences for tomatoes vary from country to country, but tomatoes with high sugar content are preferred in various countries. Therefore, sugar content measurement has been used for quality evaluation, and near-infrared (NIR) measurement devices have recently become popular as a non-destructive measurement of sugar content [5,6], which is replacing the traditional Brix refractometer, a destructive measurement device for tomato quality evaluation [7]. However, the device requires calibrating the NIR values for each measurement, depending on the cultivars, growing methods, and production locations [8]. Therefore, there is a demand to reduce this calibration as much as possible at the post-harvest facilities and distribution. It would be ideal if all measurements could be made in a one-time calibration.

Some varieties of cherry tomatoes have very high sugar concentrations [9]. Additionally, it is known that the Brix% of cherry tomato fruits could be varied [10] in cultivars, growing methods, harvest timing, and other factors. Smaller currant tomatoes are also available on the market [11], but the relationship between Brix% values and NIR values has not been fully investigated [7]. It is also important for the quality evaluation and production management to simulate a relationship between fresh weight and Brix% among tomatoes with different cultivars, etc.

In this paper, therefore, the relationship between Brix% values and NIR values was examined for cherry and currant tomatoes in addition to medium tomatoes with larger sizes; the tomatoes had different cultivars, growing methods, harvest timing, production locations, and sizes. Additionally, the relationship between fresh weight and Brix% was modeled.

## 2. Materials and Methods

### 2.1. Plant and Fruit Materials

Cherry tomato plants, *Solanum lycopersicum* L. cv. TY Chika, which produces red-colored fruits and currant tomato plants, *Solanum pimpinellifolium* L. cv. Microbeads, which also produce red-colored fruits, were tested in this study. Plants were grown hydroponically in a greenhouse. Cherry tomato ‘TY Chika’ and currant tomato ‘Microbeads’ were planted on 11 November 2021 and 2 November 2022, respectively. Ripe fruits were harvested from plants. Harvests were made from 10 plants for ‘TY Chika’, and 8 plants from ‘Microbeads’. Additionally, tomatoes from markets and supplemental local sources (hereafter, M&S) were also measured. Production area and growing methods vary for market-purchased tomatoes. The M&S tomatoes were also separated into two categories: (1) cherry tomatoes and others or (2) non-cherry tomatoes. We noted that growing methods were unknown for market-purchased tomatoes, and production locations were not identified for some commercial products. All M&S tomatoes were red in color.

### 2.2. Growing Conditions

‘TY Chika’ and ‘Microbeads’ were hydroponically grown in a north–south oriented greenhouse in Takasaki University of Health and Welfare, Takasaki, Gunma Prefecture, Japan (36°19′32.7″ N, 139°03′24.3″ E). Width, length, eaves height, and ridge height are 9 m, 21 m, 4 m, and 6.2 m, respectively. The OAT-A formula, using OAT House 1 and 2 (OAT Agrio Co., Ltd. Tokyo, Japan), was used as hydroponic solution. The target EC for fully grown plants was 2.0 dS m^−1^. Tomato plants were grown on high-wire systems. Hydroponic solution was provided through arrow drippers to rockwool blocks. Further specifications of the smart greenhouse can be found in Omasa et al. (2022) [12].

### 2.3. Measurements

#### 2.3.1. Basic Characteristics

Briefly after the harvest, the fresh weight (FW), polar (PD) (mm), and equatorial (ED) (mm) diameters of the fruits were measured using an electronic balance and digital venire calipers. Fruit shape index (SI) is calculated as PD/ED. It is the ratio of the maximum height length to maximum width of a tomato. Similarly, M&S tomatoes obtained from markets and supplemental local sources were analyzed right after the acquisition.

#### 2.3.2. NIR Spectroscopy and Soluble Solids Content (SSC) Measurements as Brix%

A desktop Near-Infrared (NIR) spectroscopy device, K-SS900LC (Kubota Corp., Osaka, Japan), was used to estimate sugar content values. The K-SS900LC is a device that measures transmittance of near-infrared light which has passed through the fruits using the interactance method. The principle of this method is to irradiate a target sample with a halogen ring light and receive the NIR-transmitted light returned from the sample to obtain an NIR spectrum. Then, a multivariate analysis is performed using the obtained second derivative spectral data as the target variable, and a calibration equation for the target component of the sample is created by comparing it with the value analyzed via the conventional destructive method, such as the digital refractometer. For the measurements, visible-NIR range of 550 to 1000 nm spectrum was used. Representative wavelengths were 842, 882, 902, and 952 nm. Wavelengths of 882 and 902 nm are related to absorption of sugar. It is considered that 842 nm is the absorption wavelength for optical path length correction. Lastly, 982 nm is the absorption wavelength of water but is thought to play a role in temperature compensation [13,14].

Four repetitions of measurements were made per fruit. An offset value for calibration was determined for value adjustment based on the average of five ‘TY Chika’ measurements using NIR value and Brix%.

Once the measurements were completed, a fruit was destructed to take juice. The juice was used to measure SSC expressed in Brix% with a digital refractometer PR-201α (Atago Co., Ltd., Tokyo, Japan). Brix% is a unit for SSC, and it is interchangeable with °Brix [15]. Fruit samples were cut in half, and juice samples were taken from two parts of cherry and M&S tomato fruits to minimize differences between sites. One part was taken from near the inner skin, and the other part from near the center. The SSC is an approximate index of the number of sugars present in fruits and vegetables. Then, a linear regression line relationship between NIR-derived Brix% value (NIR value) and refractometer-based Brix% (Brix%) was determined using the ordinary least square methods.

#### 2.3.3. Currant Tomato Fruit Measurements

In case of currant tomato ‘Microbeads’ fruits measurements, an additional procedure was needed since the fruits were so small. For NIR measurements, a black cloth cover was used to prevent interference from stray light. Once NIR measurements were performed, the fruits were crushed in a mortar and pestle to form puree. Then, puree samples were centrifuged at 3780× *g* for 10 min using a CN-2060 centrifuge (AS ONE Corp., Osaka, Japan) in microtubes. Lastly, supernatants were collected using a pipette for digital refractometer measurements.

## 3. Results

### 3.1. Basic Fruit Characteristics

In this study, basic fruit characteristics data were first measured. Table 1 summarizes basic fruit characteristics based on mean values and min-max ranges. Large differences from 4.0% to 14.2% for Brix% and 1.25 g to 95.84 g for fresh weight were observed among all tomato fruit samples. Hydroponically grown cherry tomato ‘TY Chika’ and currant tomato ‘Microbeads’ fruits and the M&S tomato fruits were used in this study. The mean values of each category of M&S tomato samples are shown on the lower side of Table 1. The average fresh weights (FW) of ‘TY Chika’, ‘Microbeads’, M&S cherry, and M&S other fruits were 10.11 g, 1.90 g, 15.00 g, and 37.20 g, respectively. Cherry tomato ‘TY Chika’ fruits showed the highest Brix% among the categories studied in this research. The Brix% of ‘TY Chika’ was 9.5% on average and had a large difference from 6.2 to 14.2% among the samples, compared with other tomato categories. A currant tomato ‘Microbeads’ showed a Brix% of 8.5% on average. The mean values in Brix% of the M&S cherry and other tomato fruits were 6.6% and 6.1%, respectively.

Round shape fruits were common for ‘TY Chika’ with an SI of 0.96 in average, while ‘Microbeads’ showed a slightly elongated fruit shape with an SI of 0.91 in average.

### 3.2. Relationships between NIR Value, Brix%, and Fresh Weight of Tomato Fruits

**Figure 1:**
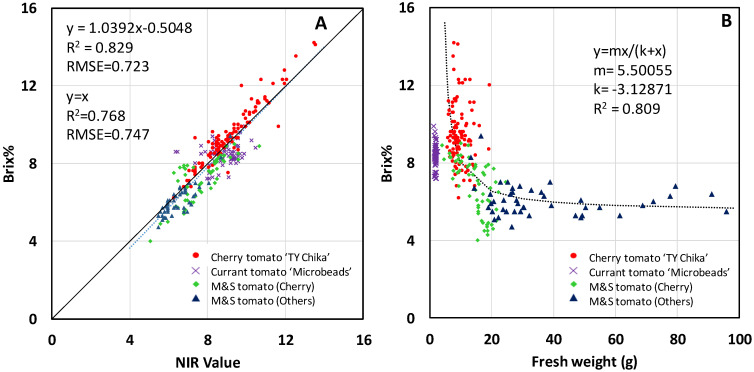
(**A**) NIR value versus Brix% of all tomato categories shown in Table 1. A dotted line shows a regression line, and a straight line shows y = x, where y is the refractometer-based Brix% (Brix%) and x is the NIR-derived Brix% value (NIR value). RMSE was 0.723 Brix% for the linear regression line and 0.747 Brix% for y = x. (**B**) Fresh weight versus the refractometer-based Brix% of all tomato categories. A dotted line shows a hyperbolic curve fit of samples except for ‘Microbeads’.

Figure 1A shows the NIR value versus Brix% of all tomato categories with various cultivars, production locations, growing methods, and sizes shown in Table 1. A linear fit explained a relationship well with a high correlation of an R^2^ with 0.829, even though the sources, varieties, growing methods, and sizes of tomatoes were different. Additionally, the Root Mean Squared Error (RMSE) was 0.723 Brix% for the linear regression line and 0.747 Brix% for x = y. Additionally, RMSEs for x = y for ‘TY Chika’, ‘Microbeads’, M&S(cherry), and M&S(others) were 0.572, 0.886, 0.896, and 0.612 Brix%, respectively. Figure 1B shows fresh weight versus Brix% of all tomato categories shown in Table 1. The lighter the fresh weight, the higher the sugar content or Brix%. A dotted line shows a hyperbolic curve fit of tomato samples, except for ‘Microbeads’, in which y = mx/(k + x), where m= 5.50055, k= −3.12871, and an R^2^ was 0.809. The hyperbolic model explains the relationship between fresh weights and Brix% well. On the hyperbolic model, cherry tomato ‘TY Chika‘ is distributed in the area with higher Brix%, the M&S cherry tomatoes are distributed around the inflection point of the figure, and the M&S other tomatoes are distributed in the right tail of the figure. The currant tomato ‘Microbeads’ data formed a cluster in the figure, which is separated from data on cherry and M&S other tomatoes.

## 4. Discussion

As shown in Table 1, the average fresh weight of cherry tomato ‘TY Chika’ is 10.11 g, which is lighter than typically expected is about 15 to 20 g for ‘TY Chika’ [16], and it also is lighter compared with 15.00 g in the mean value for M&S cherry tomatoes. The currant tomato ‘Microbeads’ fruits showed an average fresh weight of 1.90 g, which is slightly heavier than that of commercially available currant tomatoes of 1.25 g [11]. The Brix% of ‘TY Chika’ was 9.5% on average and had a large difference from 6.2 to 14.2% among the samples compared with other tomato categories. The value is higher than the values of regular tomato fruits and also higher than typical cherry tomato fruits [4]. In this study, the fresh weight of ‘TY Chika’ was lighter than usual. This might be affected by the Brix% of ‘TY Chika’. Roohanitaziani et al. (2020) [3] measured the Brix% of 107 accessions of tomatoes, and Brix% varied from 3.5 to 9.8 in the 2013 growing season and 3.2 to 11.2 in the 2014 growing season. The variation in this research could be based on tomato accessions and production year. There are also additional factors, such as growing methods, growth conditions for each fruit, and harvest timing, which affect Brix%.

There is little information on the effectiveness of commercial near-infrared (NIR) spectrometers when used with different sizes of tomatoes, which include currant tomatoes [11]. In Figure 1A, a regression line was obtained to model the relationship between the NIR value and the traditional Brix% measurements. The regression line y = 1.0392x − 0.5048 explained the relationship well as R^2^ of 0.829 and an RMSE of 0.723Brix%, regardless of the diversity of the fruits, such as cultivars, growing methods, harvest timing, and production locations. An RMSE for x = y was 0.747Brix%. The slope of the regression line was close to y = x and little difference in both RMSEs. Therefore, it was revealed that we can practically estimate Brix% for these samples using y = x without the use of the regression line after only a one-time offset calibration of the value measured with NIR spectrometers.

Some research has indicated that there might be an inverse relationship between fresh weight and Brix%. For example, Rudich et al. (1977) [1] showed a linear inverse relationship between yield and soluble solids contents (SSC) of tomato fruits. Roohanitaziani et al. (2020) [3] also showed inverse relationships between fruit weight and Brix%. However, this study did not model the relationships. In this study, an inverse relationship between fresh weight and Brix% can be modeled using a hyperbolic curve fit, and the model showed an R^2^ of 0.809 except for ‘Microbeads’, as shown in Figure 1B. In Figure 1B, a large difference in Brix% was observed for cherry tomato ‘TY Chika’ even under similar fresh weight. The potential cause of this difference could be due to differences in the growing environment, harvest timing, individual plant characteristics, location of the fruit bunch in a plant, and location of the fruits within the fruit bunch. Data distribution of cherry tomato groups such as ‘TY Chika’ and M&S cherry tomatoes was closer, indicating a roughly linear correlation between fresh weight and Brix%. For currant tomato ‘Microbeads’, the fresh weight and Brix% did not change much compared with ‘TY Chika’ and formed a cluster, which can be easily separable from data on cherry tomatoes and the M&S as ‘Microbeads’ and ‘TY Chika’ are different species. Additionally, growth and harvest periods are different. Further research may be needed on this subject. Other types of tomatoes in the M&S showed a long tail on the right-hand side of the figure, showing similar Brix% at different fresh weights. The reason for this is uncertain, but one possible reason is that there may be some sort of threshold within the samples. Overall, a hyperbolic curve fit determined in this study explained the relationship between fresh weight and Brix% well, except for currant tomato ‘Microbeads’. While the number of cultivars and growth conditions are still limited, this kind of knowledge can be used to manage tomato production and evaluate the quality. Based on this knowledge, one may be able to predict the Brix% of tomatoes, which is an important factor in the taste of tomatoes.

## 5. Conclusions

In this study, the nondestructive commercial near-infrared (NIR) spectroscopy to estimate Brix% was verified using all samples of cherry tomato ‘TY Chika’, currant tomato ‘Microbeads’, and M&S tomatoes. Additionally, the relationship between fresh weight and Brix% of all samples was examined. These tomatoes had a diversity of cultivars, growing methods, harvest timing, and production locations and varied widely from 4.0% to 14.2% for Brix%. Regardless of the diversity of samples, it was revealed that the refractometer-based Brix% (y) was practically estimated from the NIR-derived Brix% value (x) using a relationship of y = x (RMSE =0.747Brix%) after only a one-time calibration for the NIR spectrometer offset.

An inverse relationship between fresh weight and Brix% can be modeled using a hyperbolic curve fit, and the model showed an R^2^ of 0.809 except for ‘Microbeads’. Cherry tomato ‘TY Chika’ fruits showed the highest Brix%. The Brix% of ‘TY Chika’ was 9.5% on average and had a large difference from 6.2 to 14.2% among the samples. Data distribution of cherry tomato groups such as ‘TY Chika’ and M&S cherry tomatoes was closer, indicating a roughly linear correlation between fresh weight and Brix%. For currant tomato ‘Microbeads’, the fresh weight and Brix% did not change much compared with ‘TY Chika’ and formed a cluster. Other types of tomatoes in the M&S showed a long tail on the right-hand side of the figure, showing similar Brix% at different fresh weights. Overall, a hyperbolic curve fit determined in this study explained the relationship between fresh weight and Brix% well, except for currant tomato ‘Microbeads’.

## Figures and Tables

**Table 1:** Basic summary of fruits characteristics based on mean, minimum, and maximum values.

		FW (g)	PD (mm)	ED (mm)	SI	Brix%	Number of Total Samples
HG(1) Cherry tomato ‘TY Chika’	Mean	10.11	24.8	25.8	0.96	9.5	110
	Min–Max	5.76–19.4	20.6–30.1	19.6–32.6	0.84–1.26	6.2–14.2	
HG(2) Currant tomato ‘Microbeads’	Mean	1.90	12.9	14.2	0.91	8.5	80
	Min–Max	1.25–2.58	10.8–15.1	11.7–16.4	0.86–1.06	7.2–9.9	
M&S tomato (Cherry)	Mean	15.00	27.9	29.0	0.96	6.6	64
	Min–Max	6.70–21.55	20.5–34.8	18.9–36.3	0.87–1.18	4.0–9.0	
M&S tomato (Others)	Mean	37.20	36.3	39.6	0.92	6.1	45
	Min–Max	13.28–95.84	25.6–48.1	28.2–56.5	0.70–1.0	4.7–9.4	
**Detail of M&S tomato** **samples**	**Mean**						
**Cherry tomatoes**							
1. Cherry tomato, Kumamoto Pref., Dec. (UC)		19.26	29.6	32.1	0.92	4.7	9
2. Cherry tomato, Kumamoto Pref., Nov (UC)		16.09	28.9	30.1	0.96	5.6	10
3. Cherry tomato, Gunma Pref. (UC) (1)		15.13	27.2	29.4	0.93	7.0	16
4. Cherry tomato (UC, UPL)		15.05	30.1	28.4	1.06	7.7	13
5. Cherry tomato, Gunma Pref. (UC) (2)		9.47	23.8	25.0	0.95	8.0	16
**Non-cherry tomatoes**							
1. Frutica, medium-sized, Gunma Pref.		60.15	42.5	48.3	0.88	5.8	10
2. Medium-sized tomato (UC, UPL)		51.84	41.9	45.1	0.93	5.9	8
3. Cocktail tomato variety (UC, UPL)		28.90	35.2	36.4	0.96	6.2	8
4. Midi-tomato (UC, UPL)		23.18	30.7	34.7	0.88	5.6	11
5. Frutica, small-sized, Gunma Pref.		21.93	31.2	33.7	0.93	7.1	8

FW: fresh weight, PD: polar diameter, ED: equatorial diameter, SI: shape index or, PD/ED, HG: hydroponically grown in a greenhouse, M&S: market purchased and supplemental tomato samples, UC: unidentified cultivars, UPL: unidentified production location.

## Data Availability

The data presented in this study are available from corresponding authors.
